# EGFR Inhibition in Non-Small Cell Lung Cancer: Resistance, Once Again, Rears Its Ugly Head

**DOI:** 10.1371/journal.pmed.0020075

**Published:** 2005-02-22

**Authors:** Jennifer Clark, Jan Cools, D. Gary Gilliland

## Abstract

Most patients with non-small cell lung cancer who initially respond to gefitinib or erlotinib (tyrosine kinase inhibitors) ultimately develop resistance and disease relapse. What is the mechanism for this resistance?

## Kinase Inhibition for Treatment of Cancer

Uncontrolled proliferation of tumor cells is a hallmark of cancer. In many types of cancer, mutations in genes that activate cellular signal transduction pathways contribute to enhanced proliferation and survival of cancer cells. One well-characterized example is mutation in tyrosine kinases, enzymes that regulate the growth and survival of cells. Tyrosine kinase activity is tightly regulated in normal cells, but is dysregulated due to mutation in some cancers, including lung cancer, resulting in enhanced proliferation and survival of cancer cells. The tyrosine kinases are attractive candidates for molecularly targeted therapy in cancer, because cancers become dependent on growth signals from the mutant tyrosine kinases. Tyrosine kinases require ATP for their enzymic activity, and thus small molecules that mimic ATP can bind to mutant kinases and inactivate them.

The paradigm for tyrosine kinase inhibition as treatment for cancer using small-molecule inhibitors was first established in the context of chronic myelogenous leukemia (CML) associated with the *BCR-ABL* gene rearrangement [1]. Imatinib (Gleevec), a 2-phenylaminopyrimidine, is a competitive inhibitor of ATP binding to the ABL kinase, thereby inhibiting the constitutively activated BCR-ABL tyrosine kinase. Imatinib induces complete remission in most patients with CML in stable phase [1], and also has activity in CML that has progressed to blast crisis [2].

Imatinib is also a potent inhibitor of the ARG, KIT, PDGFRA, and PDGFRB tyrosine kinases. As a consequence, there have been additional dividends from the United States Federal Drug Administration approval of imatinib for treatment of BCR-ABL-positive CML. For example, imatinib is effective in treatment of chronic myelomonocytic leukemia with gene rearrangements that constitutively activate *PDGFRB* [3], of hypereosinophilic syndrome with activating mutations in *PDGFRA* [4], and of gastrointestinal stromal cell tumors associated with activating mutations in *KIT* [5] (all reviewed in [6]).

More recently, this paradigm has been extended to treatment of non-small cell lung cancer (NSCLC). Several mutations have been identified in the context of *epidermal growth factor receptor (EGFR)* in patients with NSCLC that are associated with clinical response to the small-molecule EGFR inhibitors gefitinib (Iressa) or erlotinib (Tarceva) [7,8,9], including in-frame deletions such as del L747–E749;A750P in exon 19, or L858R in exon 21. Although responses are often dramatic, most responding patients ultimately develop clinical resistance and relapse of disease [7,8,9]. The basis for resistance had not been known, in part owing to the difficulty in obtaining tissue from re-biopsy at time of relapse.

## Resistance to Small-Molecule Tyrosine Kinase Inhibitors

As might have been anticipated in treatment of cancer with any single agent, resistance to small-molecule tyrosine kinase inhibitors has emerged as a significant clinical problem. This was first appreciated in patients with CML treated with imatinib whose tumors developed resistance, and has been most extensively studied in that context. Although there are many potential mechanisms for development of clinical resistance, most cases of imatinib-resistant CML are due to point mutations in the *BCR-ABL* kinase domain itself, including T315I [10,11]. Similar mutations in the homologous residues of the kinase domains of PDGFRA (T674I) and KIT (T670I) account for imatinib resistance in some patients with hypereosinophilic syndrome and gastrointestinal stromal cell tumors, respectively [4,12]. These findings suggest strategies to overcome resistance that include the use of alternative small-molecule inhibitors. Indeed, about three years after the recognition of imatinib resistance mutations in BCR-ABL-positive CML, new drugs are now in clinical trials that are potent inhibitors of imatinib-resistant BCR-ABL mutants [13,14].

## A Basis for Resistance to Small-Molecule EGFR Inhibitors in NSCLC

In an elegant new study in *PLoS Medicine*, Pao and colleagues have identified acquired mutations in patients with NSCLC that appear to explain clinical resistance to gefitinib or erlotinib [15]. The mechanism of resistance in three patients was acquisition of a T790M substitution in EGFR that was not present at time of diagnosis, but was detected with progression of disease after initial response to gefitinib or erlotinib. T790M in the context of either transiently expressed wild-type EGFR or the mutant alleles del L474–E749;A750P or L858R impairs inhibition by gefitinib or erlotinib as assessed by autophosphorylation. Furthermore, the NSCLC cell line H1975 harbors both the L858R and T790M mutations, and is resistant to inhibition by gefitinib or erlotinib, unlike cell lines that express the L858R allele alone. In the H1975 cell line, it was possible to obtain adequate quantities of RNA to confirm that the L858R and T790M mutations are present on the same allele, as would be predicted if T790M confers resistance to inhibition of the L858R allele.

Structural models of EGFR provide structural insights into these biological data. A ribbon structure of erlotinib bound to the EGFR kinase domain (Figure 1) shows the threonine residue at position 790 in green and the positions of the exon 19 and L858R gain-of-function mutations. Substitution of methionine for threonine at position 790 would be predicted to result in steric hindrance of erlotinib binding to EGFR (Figure 2).

**Figure 1:**
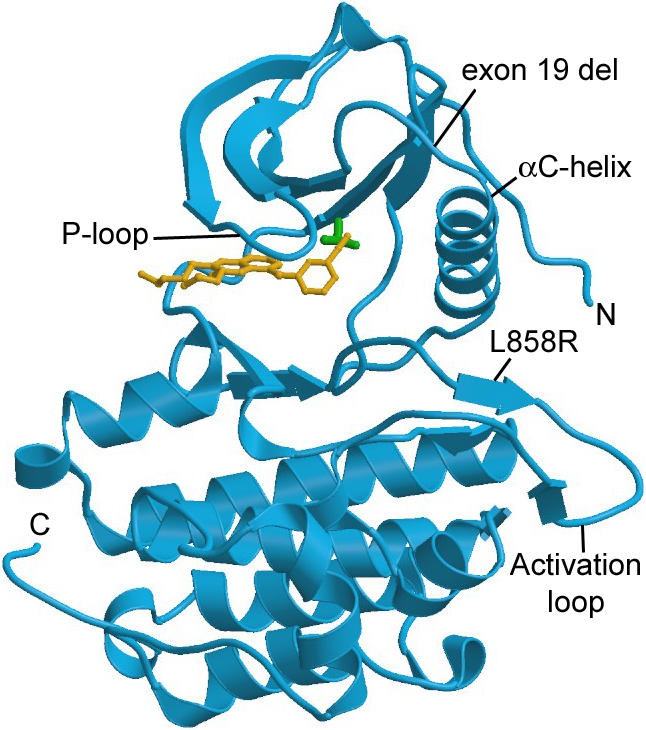
Erlotinib Bound to the EGFR Kinase Domain Schematic representation of the wild-type EGFR tyrosine kinase domain (cyan) bound to erlotinib (orange) from the Protein Data Bank (http://www.rcsb.org/pdb/) entry 1M17. The threonine 790 side chain is shown in green. The positions of the phosphate-binding loop (P-loop), the αC-helix, and the activation loop (conserved structural features in kinase domains) are shown for reference. Sites of common lung-cancer-associated drug-sensitive mutations (exon 19 deletion [del] and L858R) are also depicted. (Figure: Nikola Pavletich, Structural Biology Program, Memorial Sloan-Kettering Cancer Center)

**Figure 2:**
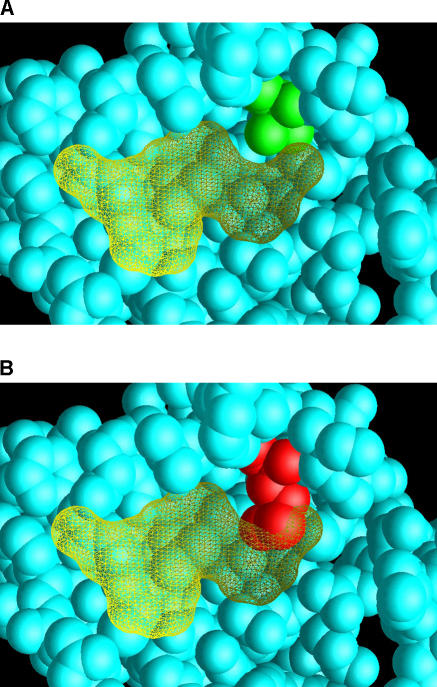
Structural Models of EGFR Showing the T790M Resistance Mutation (A) Space-filling representation of the wild-type kinase active site (cyan) with the viewer looking down the vertical axis. The structure above the plane of the figure is omitted for clarity. The threonine 790 side chain is green, and erlotinib's molecular surface is shown as a yellow net. (B) The threonine 790 side chain is replaced by the corresponding methionine side chain from the structure of the insulin receptor tyrosine kinase (Protein Data Bank entry 1IRK). The EGFR and insulin receptor have a similar structure in this region of the active site. The methionine side chain would sterically clash with erlotinib, as shown, as well as with the related kinase inhibitor gefitinib (not shown). (Figure: Nikola Pavletich, Structural Biology Program, Memorial Sloan-Kettering Cancer Center)

These observations provide convincing evidence that, at least in some patients with NSCLC, resistance to gefitinib or erlotinib can be attributed to acquisition of a T790M mutation in the context of EGFR. However, three additional patients with clinical resistance to gefitinib or erlotinib did not have the T790M mutation, nor did they have mutant *KRAS* alleles that have previously been shown by these same authors to confer resistance to these inhibitors [9]. Thus, mechanisms of resistance are heterogeneous.

## Next Steps, and Lessons Learned

It will be important to identify alternative small-molecule inhibitors for the T790M resistance mutation. Structural data suggest that one compound, lapatinib, may subserve this purpose [16], but it has not been tested for biological activity in this context. New chemical screens and/or rational drug design to identify alternative inhibitors is warranted. In addition, only half of this small cohort of patients with NSCLC with clinical resistance to gefitinib or erlotinib had the T790M substitution. Efforts to identify alternative mechanisms for resistance may be guided by experience with imatinib resistance in the context of BCR-ABL, and should include full-length sequencing of EGFR to identify other resistance mutations, and analysis for evidence of gene amplification, as well as investigation of other well-characterized mechanisms of drug resistance such as drug efflux or increased drug metabolism.

Pao and colleagues' superb study also highlights several important points that may guide development of kinase-targeted therapies in the future. It is clear that, to the extent that small-molecule kinase inhibitors are effective as single agents in treatment of cancer, resistance will develop. Furthermore, based on previous experience, some of these patients are likely to harbor acquired point mutations in the target kinase that confer resistance. Resistance mutations identified via in vitro screens have shown a high degree of correlation with those that develop in vivo, as shown in screens for imatinib-resistant BCR-ABL mutants [11] and PKC412-resistant FLT3 mutants [17], as well as the T790M resistance mutation to gefitinib in the context of EGFR [18]. Thus, in vitro screens for mutations that confer resistance to kinase inhibitors are warranted, followed by efforts to identify drugs that overcome resistance. This proactive approach should shorten the time frame for new drug development.

These findings also emphasize the critical need for re-biopsy of patients with cancer treated with molecularly targeted therapies at time of relapse. Tissue acquisition is more challenging in solid tumors than for hematopoietic malignancies, and may entail risk. Nonetheless, it is clear that data derived from such analyses will be essential to inform approaches to improving therapy for NSCLC and other solid tumors.

## Abbreviations

CML: chronic myelogenous leukemia
*EGFR*: *epidermal growth factor receptor*
NSCLC: non-small cell lung cancer
